# Cellular and Immunotherapy for Pediatric Brain Tumors: A Primer

**DOI:** 10.3390/curroncol33090522

**Published:** 2026-08-31

**Authors:** Irem Yenidogan, Nirav Thacker, Anirban Das, Magimairajan Issai Vanan

**Affiliations:** 1 Division of Hematology-Oncology, The Hospital for Sick Children, Toronto, ON M5G 1X8, Canadaanirban.das@sickkids.ca (A.D.); 2 Hematology-Oncology, Children’s Hospital of Eastern Ontario, Ottawa, ON K1H 8L1, Canada; nthacker@cheo.on.ca; 3 Division of Hematology-Oncology, Cancer Care Manitoba, Winnipeg, MN R3E 0V9, Canada

**Keywords:** immunotherapy, CAR T cells, immune checkpoint inhibition, oncolytic viruses

## Abstract

Brain tumors are the most common solid malignancies in children and the leading cause of cancer-related mortality in this age group. Cancer immunotherapy has shown a lot of promise in the treatment of both adult and pediatric cancers. In this review, we summarize the use of immunotherapies in the treatment of pediatric brain tumors with special emphasis on CAR T cells, Immune checkpoint inhibitors and oncolytic viral therapy.

## Simple Summary

Brain tumors are the most common solid cancer in children and the leading cause of childhood cancer deaths. While these tumors are difficult to treat, immunotherapy—a treatment that helps the body’s own immune system fight cancer—is showing great promise for young patients. The three main modalities of immunotherapy that will be discussed in this review include (a) CAR T cells: Doctors re-engineer a patient’s own immune cells in a lab to find and destroy cancer cells. (b) Immune Checkpoint Inhibitors: These drugs take the “brakes” off the immune system so it can see and attack hidden cancer and (c) Oncolytic Viral Therapy: Scientists use safe, modified viruses to infect and kill cancer cells directly while waking up the immune system. In short, this review highlights how medical experts are successfully using these three innovative, immune-boosting therapies to target and destroy pediatric brain tumors more effectively.

## 1. Introduction

Immunotherapy for brain tumors in its broad definition includes all types of treatments that harness the immune system of the host to attack malignant brain tumor cells and includes antibodies (e.g., Bispecific T cell engagers [BITEs]), Immune Checkpoint inhibitors (ICIs), cancer vaccines, oncolytic viral therapy and chimeric antigen receptor T-cell (CAR T cell) therapy [1,2,3]. In addition to conventional therapies like chemotherapy, radiation and targeted therapies, immunotherapies have shown the ability to eradicate cancer cells and generate long-lasting responses in human cancers. Some of the major hurdles in the clinical application of immunotherapy for brain tumors include the low blood–brain barrier (BBB) penetrance and heterogeneity with limited tumor antigen specificity (CAR T cell therapy), lower mutational burden in pediatric tumors compared to adults (ICI therapy) and increased risk of neurotoxicity with difficulties in monitoring. In this review we will summarize recent developments in adoptive cellular therapy (CAR Ts) and active immunotherapies (ICI and oncolytic virotherapies) for the treatment of pediatric brain tumors.

## 2. Adoptive Cellular Therapy: CAR T Cell Therapy

### 2.1. Biology

Chimeric Antigen Receptors (CAR T) cells are a form of adaptive cellular therapy in which the T-cells are genetically modified to express CARs on their surface [4,5,6]. Conventional cellular immune response requires the surface receptor on the T-cells to recognize antigen bound to proteins encoded by the major histocompatibility complex (MHC). The level of T-cell activation following the antigen binding is controlled by interaction of different co-stimulatory receptors such as CD28 and cytokine signaling. Cancer cells frequently downregulate MHC expression to avoid T-cell recognition and immune-mediated killing. In CAR T cells, the extracellular antigen-recognition domain is engineered to allow for the recognition of a target antigen without presentation by the MHC [4,5,6]. This extracellular antigen recognition domain (single-chain variable fragment, scFv) is genetically engineered to contain the light (VL) and heavy chain (VH) variable domains derived from a monoclonal antibody against specific tumor antigens and are specific for a given antigen expressed by a tumor cell (tumor-associated antigen, TAA). In the classic T-cell activation, once the T-cells engage the antigen, the antigen presenting cell provides co-stimulatory signals leading to cytolytic activity, proliferation of the T-cells and persistence in the tumor microenvironment. In the genetically engineered CAR T cells, the design of the intracellular domain by addition of various co-stimulatory molecules like CD28 or CD137 can improve killing efficiency, increase persistence and prolong treatment response [4,5,6,7].

Based on the intracellular domain components, CAR T cells have evolved over time with improved versions over multiple generations (1st to 5th generation CAR T cells) [4]. The modifications in the co-stimulatory domain are driven by increased anti-tumor activity, demonstrated by chemokine release and increased proliferation, and the persistence of the CAR T cells in the tumor microenvironment leading to prolonged duration of response [4,5,6,7].

### 2.2. Development and Administration

Currently most CAR T cells used in the clinic are produced using the patient’s own cells, thus avoiding the risk of graft-versus host disease. There are multiple steps in the production of autologous CAR T cells including leukapheresis followed by T-cell enrichment. Subsequently the purified T-cells are stimulated to proliferate and undergo in most cases viral transduction (retroviral or lentiviral vectors) with the vectors carrying the CAR-encoding message of the TAA transcribed and expressed as a CAR construct on the T-cell surface [5,6,8]. The newly produced CAR T cells then undergo expansion and quality control, after which the cells are infused back into the patient. The Food and Drug Administration (FDA) has also authorized clinical trials of genetically modified allogeneic CAR T cells [8].

Currently CAR T cells are infused intravenously as a single bolus into the patient for non-CNS neoplasms; however, there is evidence in the literature to suggest that locoregional infusions (including intra-cerebroventricular (ICV) or intra-tumoral (IT) of multiple small doses are well tolerated, effective and have less systemic toxicity [7].

### 2.3. Clinical Applications of CAR T Cell Therapy in Brain Tumors

CAR T cell therapy (anti-Cd19 CAR T) has been shown to provide durable remission and prolonged survival in CD19+ relapsed or refractory B-cell malignancies, including B-cell leukemias and non-Hodgkin’s lymphomas [9]. However, there are major hurdles that block the efficacy of CAR T-cell therapy in solid tumors including TAA specificity, TAA escape, CAR T cell trafficking into the tumor, proliferation and cytolytic activity of CAR T cells in the immunosuppressive tumor microenvironment (TME) and CAR T cell therapy-associated toxicity [10]. The BBB poses an additional challenge in the treatment of brain tumors. Currently, brain tumors are the most common solid tumors for clinical trials with CAR T cell therapy. The commonly used TAAs for CAR T cell therapies (Table 1) and ongoing clinical trials of CAR T cell therapies in pediatric brain tumors (Table 2) are listed in the tables.

### 2.4. CAR T Cell Therapy Related Challenges/Toxicities

One of the major causes of nonresponse and relapse after CAR T cell therapy is exhaustion. Exhausted CAR T cells have impaired anti-tumor activity and less proliferative capacity. Some of the causes of exhaustion include persistent antigen stimulation, tonic signaling from the CAR structure and immunosuppressive TME. Several modifications to overcome exhaustion have been tried including better CAR design (to prevent tonic signaling and constant antigen stimulation) along with combination therapy of CAR T with ICI therapy (PD-1 blockade) [22,23,24].

The activation of the CAR T cells within the tumor causes an inflammatory response and causes tumor cell lysis. However, the inflammatory response in the brain can lead to acute life-threatening toxicities and death. Some of the commonly encountered toxicity syndromes like Cytokine release syndrome (CRS), Immune effector cell-associated neurotoxicity syndrome (ICANS) and Tumor inflammation-associated neurotoxicity (TIAN) and their management are reviewed in these references [7,22,25,26,27].

### 2.5. Future Directions

Although CAR T cell therapy has been extremely promising in lymphoid malignancies, the field has not reached its full potential in treating solid tumors. There are several studies reporting exciting, complete or major responses in a subset of brain tumor patients leading us to believe in the promise of CAR T cell therapy in CNS tumors. Some of the areas of debate include route of administration (Intravenous (IV) vs. ICV vs. IT) and the type of immune response elicited, T-cell trafficking into the tumor, activation and persistence, TAA selection (i.e., multiple antigen CARs) and management of toxicities will be solved by multi-institutional collaborations and consensus guidelines.

In summary, CAR T cell therapy for pediatric brain tumors holds enormous promise in the future for a prolonged response and a possible cure. However, given the financial implications of producing CAR T cells per patient, the therapy is not easily available in vast areas of the world. Improvement in technology will hopefully mitigate this inequity in access to CAR T cell therapy. The challenges and unanswered questions in this field and the possible strategies to overcome them with the latest up-to-date information on the ongoing trials are addressed in these excellent reviews [23,24].

## 3. Active Immunotherapy

### Immune Checkpoint Inhibitors (ICI)

Immune checkpoint inhibitors (ICIs) have fundamentally redefined the landscape of cancer therapy in adults with extracranial malignancies. ICIs restore antitumor immunity by dismantling distinct tolerance mechanisms: antagonizing CTLA-4 to enhance T-cell priming within secondary lymphoid tissues, and disrupting the PD-1/PD-L1 axis to reverse effector T-cell exhaustion within the TME [28]. However, the successful integration of ICIs into pediatric CNS neuro-oncology has been challenging. The initial findings from Phase I/II clinical trials involving ICI monotherapy in unselected refractory pediatric CNS malignancies revealed generally low response rates [29]. The disappointing outcomes are attributed to the low tumor mutational burden (TMB) and immune-suppressed TME that characterizes the majority of pediatric brain tumors [30].

In contrast, the need for careful, biomarker-driven selection of patients for ICI treatment has been exemplified by the dramatic clinical success observed in pediatric CNS tumors defined by DNA mismatch repair deficiency (MMRd) [31,32,33]. This is rooted in the unique immunological configuration of the MMRd TME. The ultra-hypermutated phenotype translates directly into the production of a substantial non-self-tumor neoantigen repertoire. This high antigenic load is directly correlated with a significantly “hotter” TME, marked by widespread infiltration of cytotoxic T-lymphocytes (CTLs), robust expression of interferon (IFN) signaling markers, and enhanced Major Histocompatibility Complex Class I (MHC-I) machinery for effective antigen presentation [31,34,35]. This heightened inflammatory and vulnerable microenvironment, often with baseline PD-L1 expression, is uniquely primed for successful blockade of the PD-1/PD-L1 axis. Clinical and registry studies have demonstrated the specific efficacy of anti-PD-1 therapy in pediatric MMRd CNS malignancies, where median overall survival ranged from 16.2 to 21.6 months and the 2-year survival rate for malignant gliomas reached 43%. Response assessment in this cohort is notably complex; while the registry identified a 64% response rate for CNS tumors, the prospective trial revealed that initial radiological progression often represented “tumor flare,” with delayed immune activity eventually driving objective response rates up to 50% [32,35]. Consequently, PD-1 inhibitors, particularly Nivolumab, have demonstrated remarkable and durable objective responses across MMRd pediatric HGGs and medulloblastomas, firmly establishing MMRd status as the paramount predictive biomarker for ICI benefit in this population [31,35,36]. Furthermore, recent comprehensive genomic analysis of 162 primary MMRd (priMMRD) gliomas has stratified these tumors into three distinct molecular subgroups, each defined by unique genetic architectures and immunogenic potentials essential for precision management. The *priMMRD-1* (ultra-hypermutant) cohort exhibits an “immune-hot” phenotype characterized by extreme mutation burden and robust CD8+ T-cell infiltration, identifying these patients as optimal candidates for upfront immunotherapy. In stark contrast, the *priMMRD-3* (*IDH1*-mutant) subgroup mimics sporadic gliomas through an “immune-cold” microenvironment and intrinsic resistance to checkpoint blockade, necessitating novel combinatorial strategies such as concurrent *IDH* and checkpoint inhibition. In between these two the *priMMRD-2* subgroup, which is typically associated with Lynch syndrome and relies on copy number alterations rather than hypermutation for tumorigenesis and probably needs combinatorial treatment approaches [37]. Similar signals of efficacy in select patients with MMRd medulloblastoma and PFA ependymoma highlight the need for development of biomarker-selected ICI use in such rare tumor entities [38,39].

It is important to reiterate that the clinical efficacy of ICIs in neuro-oncology except for MMRd gliomas is still limited. While there are promising preclinical data for targeting non-traditional checkpoints beyond PD-1/PD-L1 and CTLA4, these are yet to be translated to clinical benefit. For example, TIM-3 has been reported to be a possible targetable checkpoint in diffuse midline gliomas [40]. Similarly preclinical data suggests efficacy for combined PD-1 and CSF1R inhibition in these deadly tumors [41]. Likewise, targeting TIGIT/CD155 was recently shown to activate natural killer cell function in some medulloblastomas. These approaches need to be investigated further through dedicated clinical trials [42].

Some of the challenges in expanding the use of ICIs for pediatric CNS tumors include the profoundly immunosuppressive and effector-sparse microenvironment of non-MMRd tumors linked to their low TMB, a lack of robust predictive biomarkers to identify tumor subsets that can still harbor high inflammatory microenvironment despite lower TMB, immunosuppressive effects of primary treatment modalities like radiation and chemotherapies, and BBB’s restriction on ICI monoclonal antibody delivery [43]. Strategies that can counter some of these challenges include use of combination treatments that enhance the immune response including dual checkpoint inhibitors, combination of demethylating agents and select oncogenic pathway inhibitors like RAS/MAPK and *IDH*-inhibitors with ICI, early use of ICI as is being tested in the neoadjuvant setting for adult glioblastoma in clinical trials, and use of novel delivery techniques like focused ultrasound to improve drug penetration across the blood–brain barrier. In this context, it is also important to note that response assessment for ICI treatment remains challenging, particularly because of the phenomenon of pseudo-progression—transient tumor enlargement due to immune infiltration—that necessitates the use of specialized Immunotherapy Response Assessment in Neuro-Oncology (iRANO) criteria to prevent the premature discontinuation of effective therapy [44,45,46]. Emerging technologies like circulating tumor DNA analysis from cerebrospinal fluid can be an attractive complement to the challenging radiology assessment for ICI in CNS tumors and needs to be systematically studied. Lastly, to manage the symptomatic edema associated with this flare while avoiding the T-cell dampening effects of corticosteroids, recent evidence supports that bevacizumab (monoclonal antibody targeting Vascular Endothelial Growth Factor, VEGF-A) can be used as a steroid-sparing intervention. Management of systemic immune-related adverse events (irAEs) also remains a challenge, as steroids remain the standard treatment, leading to potential dampening of oncologic efficacy of ICIs, suggesting a need to explore steroid-sparing or limiting strategies.

In summary, while ICIs have proven transformative for MMRd CNS tumors, broader efficacy in other tumor subsets necessitates a highly nuanced, biomarker-driven approach, development of combinatorial strategies targeting the genomic drivers and microenvironment targets beyond traditional immune checkpoints, improving drug delivery, as well as response assessment and side effect management strategies in patients. A comprehensive overview of clinical trials, registry cohorts, and case reports evaluating the efficacy of ICIs in pediatric CNS tumors is detailed in Table 3.

## 4. Oncolytic Viruses (OV)

Oncolytic virotherapy is an emerging therapeutic strategy in oncology that utilizes naturally occurring or genetically engineered viruses capable of preferentially infecting and replicating within malignant cells [56]. Unlike passive immunotherapies, oncolytic viruses (OVs) function through a dual mechanism known as viro-immunotherapy: they provide direct tumor cytolysis (“virological debulking”) and act as a biological “spark” to trigger systemic antitumor immunity. This dual functionality is particularly relevant in pediatric central nervous system (CNS) tumors, which are typically characterized by low tumor mutational burden and relatively immunologically quiescent tumor microenvironments. These biological features have historically limited the effectiveness of conventional immune-based therapies. OVs therefore offer a strategy to reshape the TME and overcome intrinsic resistance.

### 4.1. Mechanism of Action

#### 4.1.1. Tumor Selective Viral Replication and Cytolysis

The therapeutic foundation of oncolytic virotherapy lies in the preferential replication of viruses within malignant cells. Tumor selectivity is achieved by exploiting oncogenic alterations that distinguish cancer cells from normal tissue. Tumors frequently harbor dysregulated signaling pathways—including abnormalities in the retinoblastoma (RB), Ras, and p53 pathways—as well as defects in antiviral defense mechanisms such as type I interferon signaling and protein kinase R (PKR) activation [57,58,59]. These alterations impair the ability of tumor cells to mount effective antiviral responses, thereby creating a permissive environment for viral replication.

#### 4.1.2. Immune Activation

Beyond direct cytolysis, OV-mediated tumor destruction initiates a cascade of immunologic events. Viral replication culminates in tumor cell death, resulting in the release of TAAs, damage-associated molecular patterns (DAMPs), and pathogen-associated molecular patterns (PAMPs). These signals alter the TME, promote dendritic cell maturation, enhance antigen presentation, and stimulate type I interferon–mediated inflammatory responses [60,61,62].

The immunostimulatory component of OV therapy is particularly important in pediatric CNS tumors, where baseline immune infiltration is typically limited. OV infection can convert an immunologically quiescent TME into a more inflamed state, facilitating recruitment of cytotoxic T cells and other immune effector populations. This immune activation may not only enhance local tumor control but may also generate systemic antitumor responses and immunologic memory that could target metastatic sites, delay or prevent recurrence [63,64,65].

### 4.2. Oncolytic Viral Platforms in Pediatric Brain Tumors

Multiple viral platforms have been investigated for oncolytic virotherapy in pediatric brain tumors. Among these, herpes simplex virus (HSV)- and adenovirus-based vectors represent the most extensively studied systems and have progressed furthest in clinical development [66]. These viruses can be engineered to enhance tumor selectivity, improve viral replication within malignant cells, and augment antitumor immune responses. Additional viral platforms—including poliovirus, reovirus, measles virus, vaccinia virus and myxoma virus—have demonstrated tumor-selective tropism and antitumor activity in preclinical models but remain at earlier stages of translational development. The major biological features and translational status of these viral platforms are summarized in Table 4.

### 4.3. Clinical Trials of Oncolytic Virotherapy in Pediatric CNS Tumors

Clinical translation of oncolytic virotherapy in pediatric neuro-oncology remains at an early stage but has demonstrated encouraging safety profiles and preliminary signals of biological activity. To date, most studies have consisted of early phase I/II trials involving children with high-grade tumors. Key clinical trials investigating oncolytic viral therapies in pediatric brain tumors are summarized in Table 5.

Among HSV-based therapies, G207 is the most extensively studied platform in pediatric patients. In a phase I clinical trial evaluating intratumoral administration of G207 combined with radiation therapy in children with recurrent or progressive high-grade gliomas, 12 patients aged 7–18 years were enrolled. Treatment was well tolerated with no severe treatment-related toxicities. Radiographic, neuropathological, or clinical responses were observed in 11 patients, and the reported median overall survival was 12.2 months (95% CI: 8.0–16.4). Four patients remained alive 18 months after treatment. Increased immune infiltration within the tumor microenvironment was also observed between two and nine months following viral administration [86].

Adenovirus-based therapies have also entered clinical evaluation. In a phase I trial investigating DNX-2401 in children with diffuse midline glioma (NCT03178032), treatment demonstrated an acceptable safety profile and encouraging survival signals, with a reported median overall survival of approximately 17.8 months, exceeding historical expectations for this disease. The dose-escalation study enrolled 12 patients aged 3–18 years who, after confirmatory biopsy, received a single intratumoral injection of Delta-24-RGD, followed by radiotherapy in 11 of the 12 patients. Tumor reduction was reported in nine patients, with partial responses in three and stable disease in eight patients [87].

A recent systematic review identified four prospective clinical trials including approximately 40 pediatric patients treated with oncolytic viruses [88]. Tumor types included glioblastoma, diffuse midline glioma, anaplastic astrocytoma, recurrent ependymoma, and diffuse hemispheric glioma. Across studies, treatment was generally well tolerated, with fever, headache, and nausea representing the most reported adverse events, while serious treatment-related toxicities were uncommon.

Although these early trials remain limited by small sample sizes and heterogeneous patient populations, they provide important proof-of-concept that oncolytic virotherapy is feasible and biologically active in pediatric CNS tumors.

**Table 5:** Clinical Trials of Oncolytic Viral therapies in Pediatric Brain Tumors.

Virus	Platform	Tumor Type	Trial Phase	Delivery Method	Key Findings
HSV-1 [86] NCT02457845	G207	Recurrent pediatric high-grade glioma	Phase I	Intratumoral injection + radiation	Favorable safety profile; immune activation observed; radiographic responses in subset of patients
HSV-1 NCT04482933	G207	Recurrent pediatric high-grade glioma	Phase II	Intratumoral injection + radiation	Funding withdrawn without single enrollment.
HSV1 NCT03911388	G207	Recurrent pediatric cerebellar high-grade tumors	Phase I	Intratumoral+ Radiation	Active, recruiting
HSV-1 (NCT02031965)	HSV1716	Recurrent/refractory pediatric brain tumors	Phase I	Intra/peritumoral injection	Trial terminated early after enrolling two patients; no formal efficacy results reported
Adenovirus [87] (NCT03178032)	DNX-2401 (Delta-24-RGD)	Newly diagnosed DIPG	Phase I	Intratumoral injection	Median OS 17.18 months; acceptable safety profile
Poliovirus [89] NCT03043391	PVSRIPO (Lerapolturev)	Recurrent Pediatric high grade CNS tumors	Phase Ib	Convection-enhanced delivery	No treatment related irreversible G4 AE or deaths. The median overall survival was 4.1 months (95% CI 1.2–10.1). One patient remains alive after 22 months.
Reovirus NCT02444546	Pelareorep	Recurrent pediatric high grade CNS tumors	Phase I	Intravenous + GM-CSF	DLT were observed in the sixth patient, and the study was closed. No complete or partial responses were observed, and all patients experienced progression within 60 days.
Measles NCT02962167	MV-NIS	Recurrent medulloblastoma or ATRT	Phase I	Intratumor or subarachnoid space	Data not published

### 4.4. Limitations and Future Directions

Despite promising early results, several challenges currently limit the clinical impact of oncolytic virotherapy in pediatric neuro-oncology.

Major barriers include restricted intratumoral viral dissemination, host antiviral immune responses that limit viral persistence, and lack of predictive biomarkers for identifying patients most likely to benefit from OV therapy. Additionally, delivery of viral therapies to CNS tumors remains technically challenging because systemic administration is restricted by the blood–brain barrier, necessitating intratumoral or convection-enhanced delivery approaches.

Future advances will likely depend on improved viral engineering, biomarker-guided patient selection, optimized delivery strategies, and rational integration with other immunotherapies such as immune checkpoint inhibitors, natural killer (NK) cell therapies, and CAR T cell therapies. Furthermore, “armed” viruses engineered to express immunostimulatory transgenes may further enhance antitumor immune responses. Collectively, these developments suggest that oncolytic virotherapy may become an important component of multimodal immunotherapy strategies for pediatric Neuro-Oncology.

## 5. Conclusions

In summary the three major immunotherapeutic strategies in the treatment of pediatric brain tumors described in this review complement one another by targeting different aspects of tumor immune evasion. CAR T-cell therapy provides highly specific cellular killing through engineered lymphocytes, immune checkpoint inhibitors reactivate suppressed endogenous T-cell responses, and oncolytic viruses simultaneously destroy tumor cells and stimulate broad immune activation. Despite promising advances, each approach faces challenges related to tumor heterogeneity, immune suppression, delivery across the BBB, treatment-related toxicities, and accurate response assessment. Current research increasingly emphasizes biomarker-guided patient selection and rational combination therapies that integrate these modalities to overcome resistance mechanisms and improve long-term outcomes.

A promising combination strategy involves administering oncolytic viral therapy before CAR T-cell therapy to enhance antitumor immune responses [63,64,65,66]. The oncolytic virus selectively infects and lyses tumor cells, releasing tumor-associated antigens and inflammatory cytokines that transform the immunosuppressive (“cold”) tumor microenvironment into an immune-active (“hot”) one. This immune priming promotes T-cell infiltration and improves the trafficking, expansion, and persistence of subsequently infused CAR T cells targeting tumor-specific antigens such as B7-H3 or GD2. By combining broad immune activation with highly specific cellular cytotoxicity, this approach may overcome poor immune infiltration and antigen recognition in pediatric brain tumors.

Combining CAR T-cell therapy with immune checkpoint inhibitors aims to enhance the durability and effectiveness of adoptive cellular immunotherapy [24,28,47]. Although CAR T cells can specifically recognize and eliminate tumor cells, their function may decline over time because of T-cell exhaustion within the immunosuppressive tumor microenvironment. Administering immune checkpoint inhibitors, such as PD-1 inhibitors, after CAR T-cell infusion can restore T-cell activity, prolong CAR T-cell persistence, and enhance sustained antitumor immunity. This combination has the potential to improve long-term therapeutic responses by overcoming immune suppression and preventing functional exhaustion of engineered T cells [24].

Collectively, these innovations represent a major shift toward precision immunotherapy and offer significant hope for improving survival and quality of life in children with brain tumors.

## Figures and Tables

**Table 1:** Common TAAs for CAR T cell therapy in pediatric brain tumors.

Antigen	Brain Tumor Expression	Expression in Normal Tissue	Function	References
B7-homolog 3 protein (B7-H3)-CD276	HGG, DMG, MBL	Expressed in normal tissues- lung, liver, bladder, testis and lymphoid organs	Increased expression contributes to tumor immune evasion, metastatic potential, poor prognosis	[11,12]
Epidermal Growth Factor Receptor (EGFR)-EGFR806	HGG, DMG, EPN, MBL, ATRT	EGFRvIII is restricted to GBM	Cell surface receptor, regulate cell growth, survival, migration.	[13]
Disialoganglioside 2 (GD2)	DMG, HGG	Brain, Peripheral nerves, skin (melanocytes)	Cell–Cell attachment to fibronectin	[14,15]
ERBB2 receptor tyrosine kinase (HER2)	GBM, EPN, MBL	Epithelial tissue, skin, muscle	Cell cycle homeostasis.	[16]
Interleukin 13 receptor subunit α2(IL-13Rα2)	HGG, GBM, MBL	Testis	Apoptosis escape mechanism	[17]
Glypican 2(GPC2)	ETMR, MBL, CNS-ET, CPC, HGG, DMG	Testis, skin	Surface oncoprotein	[18]
CD70	HGG, GBM	Activated B, T cells NK and Dendritic cells	Increased expression promotes stemness, invasion, migration.	[19,20]

ATRT—Atypical Teratoid Rhabdoid Tumors, CNS-ET—Central nervous system Embryonal tumors, CPC—Choroid Plexus Carcinomas, ETMR—Embryonal tumor with multilayered rosettes, DMG—Diffuse Midline Gliomas, EPN—Ependymomas, GBM—Glioblastoma Multiforme, HGG High Grade Gliomas, and MBL—Medulloblastoma. Table adapted and modified from Ref [6,21].

**Table 2:** Current clinical trials of CAR T cell therapy for Pediatric brain tumors.

Clinical Trial Number (NCT#)	Title	Phase	Target	Delivery	Eligible Tumors	Status
06221553	Safety and Efficacy of Loco-regional B7H3 IL-7Ra CAR T Cell in DIPG (CMD03DIPG)	1	B7H3	ICV	DMG (DIPG)	Recruiting
06946680	IL-8 Receptor-modified CD70 CAR T Cell Therapy in CD70+ Pediatric High-grade Glioma (HGG) (IMPACT)	1	CD70	IV	pHGG	Recruiting
04099797	C7R-GD2.CAR T Cells for Patients with GD2-expressing Brain Tumors (GAIL-B)	1	GD2	IV, ICV	DMG, HGG, MBL, ATRT, EPN	Recruiting
05298995	GD2-CAR T Cells for Pediatric Brain Tumors	1	GD2	IV	HGG, DMG, MBL, ATRT	Recruiting
07087002	GPC2-CAR T Cell Therapy for Relapsed or Refractory Medulloblastoma in Children and Young Adults	1	GPC2	ICV	MBL, ATRT, ETMR, CNS-ET, Pineoblastoma	Recruiting
04510051	CAR T Cells After Lymphodepletion for the Treatment of IL13Rα2 Positive Recurrent or Refractory Brain Tumors in Children	1	IL-13Rα2	ICV	HGG, GBM, MBL	Recruiting
04661384	Brain Tumor-Specific Immune Cells (IL13Ralpha2-CAR T Cells) for the Treatment of Leptomeningeal Glioblastoma, Ependymoma, or Medulloblastoma	1	IL-13Rα2	ICV	Leptomeningeal GBM, MBL, EPN	Active, Not recruiting
05768880	Study of B7-H3, EGFR806, HER2, And IL13-Zetakine (Quad) CAR T Cell Locoregional Immunotherapy for Pediatric Diffuse Intrinsic Pontine Glioma, Diffuse Midline Glioma, And Recurrent or Refractory Central Nervous System Tumors	1	B7-H3, EGFR806, HER2, and IL-13-zetakine	ICV	DIPG, DMG, recurrent/refractory brain tumors	Recruiting
ACTRN12622000675729 (*)	A Phase I Study of the Safety of Autologous GD2-Specific Chimeric Antigen Receptor-Expressing T Cells in Children with Diffuse Midline Glioma	1	GD2	IV	DMG, DIPG, HGG	Recruiting

ATRT—Atypical Teratoid Rhabdoid Tumors, CNS-ET—Central nervous system Embryonal tumors, CPC—Choroid Plexus Carcinomas, ETMR—Embryonal tumor with multilayered rosettes, DMG—Diffuse Midline Gliomas, EPN—Ependymomas, GBM—Glioblastoma Multiforme, HGG High Grade Gliomas, MBL—Medulloblastoma. ICV—Intra-Cerebro Ventricular, IV—Intravenous. For all other abbreviations, please see Table 1. * = Rare Cancers Australia clinical trials registry. (All data are up to date as of 15 July 2025).

**Table 3:** Clinical trials of ICI therapy in pediatric brain tumors. (adapted from Yenidogan et al. 2026, Table 2, [47].

Trial/Study (NCT)	Phase	Tumor Types	Agent(s)	Efficacy	Status
NCT02332668 2015–2027	Phase I/II	Advanced melanoma; PD-L1+ relapsed/refractory solid tumors and lymphoma; small subset with CNS involvement	Pembrolizumab	A separate MSI-H cohort of KEYNOTE-51 enrolled 7 pediatric patients (6 with CNS malignancies), one HGG patient achieved durable complete response after initial progression (at cycle 6, maintained cycle 20)	Ongoing long-term follow-up
NCT02359565 2015–2025	Phase I	High-Grade CNS tumors	Pembrolizumab	Early results-2018 ISPNO: 5 patients with DIPG progressed more rapidly than historical controls with a median PFS of 1 month, prompting an amendment to the study to exclude recurrent DIPG.	Completed, results not posted
NCT02541604 2015–2019	Phase 1/2	Solid Tumors	Atezolizumab	4 of ~87 (about 5%) achieved even a partial response by 6 months	Completed; contributes pharmacokinetic and biomarker data for pediatric atezolizumab
NCT02793466 2016–2023	Phase I	Solid Tumors, Lymphoma	Durvalumab monotherapy ± chemotherapy/RT in disease- and age-defined cohorts	3 of 5 ependymoma patients had clinical benefit and one achieved a formal RECIST partial response	Completed, supports further biology-driven studies rather than broad use
NCT02992964 2017–2023	Phase 1/2	Hypermutant solid and CNS tumors	Nivolumab	Response rate rose from 20% at first assessment to 50% with longer follow-up, and 4 patients—3 with malignant glioma—remained in complete remission at a median 37 months	Completed, reinforces TMB/MMRd as the operative selection criterion
NCT03130959 2017–2022	Phase 1b/2	High-grade CNS tumors	Nivolumab monotherapy vs. nivolumab + ipilimumab	Median OS in newly diagnosed DIPG was 11.7 months (nivolumab) and 10.8 months (combination)	Completed; negative trial for unselected pediatric CNS tumors; informs design of biomarker-enriched studies
NCT04323046 2020–2029	Phase 1	Recurrent/ progressive CNS tumors	PD-1/PD-L1-based immunotherapy regimen before/after Surgery	Results pending	Ongoing; will clarify role of next-generation PD-1/PD-L1 strategies in pediatric CNS tumors
**Registry and Cohort Studies**					
**Study**	**Phase**	**Tumor types**	**Agent(s)**	**Efficacy**	**Status**
Das e al. 2022 [35]	International Consortium Registry Study	MMRD and PPD cancers	Nivolumab, Pembrolizumab	41.4% 3-year survival (*n* = 45); TMB and MS-indel burden predict response via distinct mechanisms	Supports further biology-driven studies
Das et al. 2024 RRD-HGG multinational cohort [48]	Retrospective multicenter cohort	RRD HGG	PD-1 inhibitor + RT/CTLA-4 inhibitor/MEK inhibitor	11.6-month post-progression survival (*n* = 38, *p* < 0.001)	Supports reirradiation continuing ICI-based salvage after first-line ICI fails
Gikandi et al. 2024 mixed pediatric CNS ICI cohort [49]	Retrospective single-/multicenter series	Hematologic malignancies, solid tumors and CNS tumors	Nivolumab/pembrolizumab ± combinations (e.g., bevacizumab, RT, chemotherapy)	irAE occurrence was associated with significantly improved progression-free survival	Supports that unselected pediatric CNS tumors rarely derive major benefit from ICI
**Case Reports**					
**Study**	**Phase**	**Tumor types (strata)**	**Agent(s)/Schema**	**Efficacy**	**Status**
Blumenthal et al. 2016 early PD-1 experience [50]	5 pediatric patients with recurrent CNS tumors	2 diffuse brainstem gliomas, 1 GB, 1 ATRT, 1 medulloblastoma.	Pembrolizumab ± bevacizumab	5 children treated with pembrolizumab, all had progressive disease; median OS was only 3.2 months in children—prompting the authors to explicitly advise against further unselected use	Negative for unselected patients
Gorsi et al. 2019 [51]	Retrospective; 10 pediatric patients	5 HGG, 1 LGG, 1 pineoblastoma, 1 medulloblastoma, 1 ependymoma, 1 CNS embryonal tumor NOS	Nivolumab	Among 10 nivolumab-treated patients, 3 had partial responses at the primary site	Negative in unselected, heavily pretreated pediatric brain tumors, with different median survival by PD-L1 status despite a very low median TMB of 1.3 mut/Mb
Cacciotti et al. 2020 (11-patient series) [52]	Retrospective series; 11 pediatric patients	DIPG [2], HGG [5], ependymoma [1], craniopharyngioma [1], high-grade neuroepithelial tumor (HGNET) [1], and NGGCT/choriocarcinoma [1]	Ipilimumab, nivolumab, pembrolizumab	In 11 patients iRANO-based responses were 3 PR, 7 SD, 1 PD, with 2 durable responders; treatment stopped for progression in 7 and for toxicity in only 2	Completed; exploratory signal that biology-enriched subsets may respond
Kline et al. 2018 DIPG nivolumab ± re-irradiation [53]	Retrospective single-institution cohort; recurrent DIPG after initial RT	31 pediatric DIPG at recurrence after initial RT (8 re-irradiation + nivolumab, 4 re-irradiation alone, 19 historical RT-only) DIPG	Concurrent re-irradiation with nivolumab followed by maintenance nivolumab vs. re-irradiation alone vs. RT-only historical cohort	Median OS was 22.9 months with reirradiation plus nivolumab vs. 20.4 months with reirradiation alone vs. 8.3 months with neither	Supports feasibility and safety of combining PD-1 blockade with re-irradiation
AlHarbi et al. 2018 [54]	Single-patient case report	supratentorial glioblastoma (WHO grade IV), CMMRD-associated	Nivolumab monotherapy	A 5-year-old female with CMMRD with 60% tumor shrinkage on nivolumab with no adverse reactions through 10 months, remaining fully ambulatory with a normal neurologic exam.	Supports germline MMRd can have ICI sensitivity
Henderson et al. 2022 ICI for Synchronous Hypermutated Cancers [55]	Two patients with synchronous, progressive, and metastatic malignancies	GBM + rectal adenocarcinoma Grade 3 Astrocytoma + Transitional cell carcinoma	Pembrolizumab, Nivolumab	Prolonged survival	Demonstrates the efficacy of immunotherapy as a rational approach for patients with CMMRD and synchronous cancers

ATRT—Atypical Teratoid Rhabdoid Tumors, CMMRD—Constitutional mis-match repair deficient, CTLA-4—Cytotoxic T-Lymphocyte-associated protein 4, DIPG—Diffuse Intrinsic Pontine Glioma, DMG—Diffuse Midline Gliomas, EPN—Ependymomas, GBM—Glioblastoma Multiforme, HGG—High Grade Gliomas, LGG—Low Grade Glioma, MMRD—Mismatch repair deficient, NGGCT—Non-germinomatous germ cell tumor, PD-1—Programmed cell death protein 1, PD-L1—Programmed cel death-ligand 1, RT—Radiation therapy, and RRD—Replication repair deficient. (All data are up to date as of 16 March 2026).

**Table 4:** Oncolytic Viral Platforms Investigated in Pediatric Brain Tumors.

Viral Platform	Representative Constructs	Key Genetic Modifications	Mechanism of Tumor Selectivity	Preclinical Evidence in Pediatric Brain Tumors
Herpes Simplex Virus (HSV-1) [66,67,68,69,70,71,72,73,74,75]	G207, HSV1716, rRp450, rQNestin, G47Delta, M032 and C134	γ134.5 deletion; UL39 inactivation; α47 deletion; cytokine transgene insertion (e.g., IL-12)	Replicates in tumor cells with impaired antiviral responses; enhanced antigen presentation and immune activation	Models of pediatric high-grade glioma, DIPG, MBL, and ATRT
Adenovirus [62,76,77,78,79,80,81]	DNX-2401 (Δ24-RGD), CRAd-S-pK7, ICOVIR17K,	E1A deletion targeting RB pathway; arginine-glycine-aspartate (RGD) peptide fiber modification to enhance integrin binding	Selective replication in RB-deficient tumor cells; improved tumor cell entry via integrin receptors	Demonstrated antitumor activity in ATRT and CNS-PNET xenograft models; MSC-mediated delivery explored in DIPG models, pediatric high-grade glioma, DIPG, ETMR
Poliovirus [82]	PVSRIPO (PV (Sabin)-Rhinovirus IRES PV Open reading frame) (Lerapolturev)	Recombinant poliovirus targeting CD155 receptor	Targets CD155 receptor	Medulloblastoma, PXA, ATRT, PNET
Reovirus [83]	Pelareorep	Naturally occurring oncolytic RNA virus	Preferential replication in tumors with activated Ras signaling	MBL
Measles Virus [84,85]	MV-CEA (human carcinoembryonic antigen), MV-NIS (sodium iodide symporter)	Engineered measles strains expressing reporter or therapeutic genes	Utilizes CD46 receptor overexpressed in tumor cells	MBL, ATRT
Vaccinia Virus	ddVV (double-deleted modified version of Vaccina virus)	Deletion of thymidine kinase (TK) and vaccinia grow factor (VGF) genes	Replicates preferentially in tumors with dysregulated signaling pathways	ATRT
Myxoma Virus		Exploits defects in antiviral signaling pathways	Selective infection of tumor cells with impaired antiviral responses	MBL

ATRT—Atypical Teratoid Rhabdoid Tumors, CNS-PNET—Central nervous system-primitive Neuro-ectodermal tumors, CPC—Choroid Plexus Carcinomas, DIPG—Diffuse Intrinsic Pontine Glioma, ETMR—Embryonal tumor with multilayered rosettes, DMG—Diffuse Midline Gliomas, EPN—Ependymomas, GBM—Glioblastoma Multiforme, HGG High Grade Gliomas, MBL—Medulloblastoma, and PXA—Pleomorphic Xantho-astrocytoma. (All data are up to date as of 16 March 2026).

## Data Availability

No new data were created or analyzed in this study.
